# Optical coherence tomography findings in methanol toxicity

**DOI:** 10.1186/s40942-017-0089-4

**Published:** 2017-09-25

**Authors:** Kendra A. Klein, Alexis K. Warren, Caroline R. Baumal, Thomas R. Hedges

**Affiliations:** New England Eye Center Department of Ophthalmology, Tufts Medical Center, Tufts University School of Medicine, 260 Tremont Street, Biewend Building, 11th Floor, Boston, MA 02111 USA

**Keywords:** Methanol, Inner nuclear layer microcysts, Optical coherence tomography (OCT), Retinal toxicity, Toxic optic neuropathy

## Abstract

**Background:**

Methanol toxicity poses a significant public health problem in developing countries, and in Southeast Asia, where the most common source of poisoning is via adulterated liquor in local drinks. Methanol toxicity can have devastating visual consequences and retinal specialists should be aware of the features of this toxic optic neuropathy. The authors report a case of severe systemic methanol toxicity and relatively mild optic neuropathy demonstrating unique retinal changes on optical coherence tomography (OCT).

**Case presentation:**

A previously healthy student developed ataxia, difficulty breathing and loss of consciousness hours after drinking homemade alcohol while traveling in Indonesia. She was found to have a serum pH of 6.79 and elevated methanol levels. She was treated with intravenous ethanol, methylprednisolone and sodium bicarbonate. When she awoke she had bilateral central scotomas. At presentation, she had central depression on visual field testing. OCT of the retinal nerve fiber layer (RNFL) was normal but ganglion cell layer analysis (GCL) showed highly selective loss of the nasal fibers in both eyes. Further, OCT of the macula demonstrated inner nuclear layer (INL) microcysts in the corresponding area of selective GCL loss in both eyes.

**Conclusions:**

The selective involvement of the papillomacular bundle fibers is common in toxic optic neuropathies and represents damage to the small caliber axons rich in mitochondria. Despite severe systemic toxicity, the relative sparing of the optic nerve in this case enabled characterization of the evolution of methanol toxicity with segmental GCL involvement and preservation of the RNFL, corresponding to the papillomacular bundle. This is the first reported case of INL microcysts in methanol optic neuropathy and supports that they are a non-specific finding, and may represent preferential damage to the papillomacular bundle.

## Background

Methanol, which is also known as wood alcohol, is a common component of antifreeze, photocopier fluid, and perfume. Although uncommon in the United States, methanol toxicity poses a significant public health problem in developing countries, and in Southeast Asia, where the most common source of poisoning is via adulterated liquor in local drinks [1]. Formic acid is a toxic byproduct of methanol metabolism, and it causes tissue hypoxia and lactic acid formation via inhibition of the mitochondrial cytochrome oxidase. Symptoms of toxicity can appear as early as 4 h after methanol ingestion and include nausea, vomiting, headache, vertigo, and confusion. Clinical signs of toxicity include increased serum methanol, metabolic acidosis, rhabdomyolysis, renal failure, shock, and death [2].

The initial ophthalmologic symptoms of methanol toxicity typically appear 6–48 h post-ingestion and severity of symptoms vary widely from mild progressive and painless decreases in vision to no-light perception vision, dyschromatopsia, scotomata, and photophobia [1]. Pupillary examination may demonstrate dilated, non-reactive pupils. Acutely, the optic disc is hyperemic and edematous, whereas chronically there is optic disc atrophy and pallor [2, 3].

The small caliber axons of the papillomacular bundle, serving central vision and rich in mitochondria, are disproportionately affected in methanol-induced optic neuropathy [4]. Microcystic changes in the inner nuclear layer (INL) have previously been described using spectral domain ocular coherence tomography (SD-OCT) as a non-specific findings associated with optic neuropathy from other etiologies [7]. We present the first case of INL microcysts on SD-OCT in a young woman with methanol toxicity and hypothesize it is related to highly specific retinal ganglion cell loss.

## Case presentation

A 19-year-old female student who had recently returned from a semester abroad in Indonesia was evaluated for bilateral blurred vision, photophobia and a central scotoma of 4 weeks duration. While in Indonesia, the night prior to symptom onset, she drank alcoholic drinks from street vendors while out with friends. She and her friends each had two shots of homemade “whiskey” with Coca-Cola. In addition, the patient ate two to three homemade chocolates containing a small amount of liquor. She was the only one to eat the liquor-filled chocolates and was the only one in the group who became ill. Later that evening, she developed nausea, vomiting, and had difficulty walking. She awoke the following day with generalized malaise and photophobia. That evening, she became increasingly somnolent and developed labored breathing. She was brought to a local emergency department where she was resuscitated and intubated for apnea. She was found to have a metabolic acidosis with a serum pH of 6.79 and a blood methanol level of 0.60 mg/dL. Urine studies failed to reveal crystals to suggest ethylene glycol poisoning. She was diagnosed with acute methanol toxicity, presumptively secondary to the liquor-filled chocolates. The exact amount of methanol consumed was not known, but it was assumed to be less than 10 mL. She was started on intravenous ethanol, methylprednisolone and sodium bicarbonate with vitamin B12. She required dialysis for treatment of metabolic acidosis.

The following day she was extubated and, upon awakening, noted bilateral blurred central vision and photophobia. In addition to the visual symptoms, she developed extremity weakness, a shuffling gait, microphonia and micrographia. Magnetic resonance imaging of the brain demonstrated classic damage to the basal ganglia associated with methanol poisoning. The T2-weighted scans revealed hyperintense lesions of the putamen bilaterally, consistent with acute hemorrhage, and generalized hyperintensity of the cerebral cortex, consistent with diffuse hypoxic injury. During her hospitalization, an ophthalmologist evaluated her and noted no structural ocular abnormalities.

**Fig. 1:**
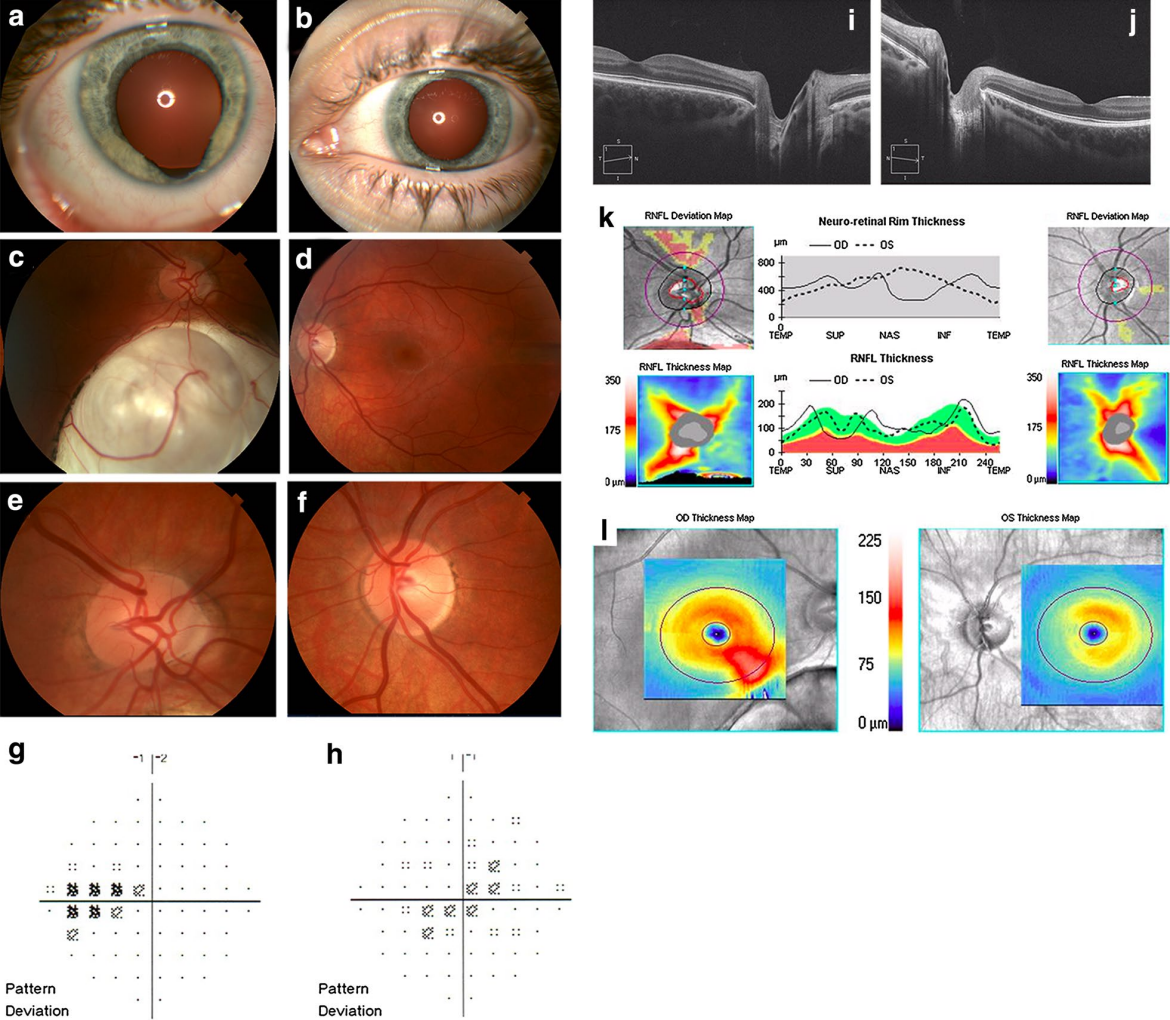
Findings at presentation. External *color photograph* demonstrating an inferonasal iris coloboma in the right eye (**a**) and a normal iris configuration in the left eye (**b**). *Color* fundus photograph demonstrating an inferonasal chorioretinal coloboma of the right eye (**c**) and a normal appearing fundus in the left eye (**d**). Optic disc photograph of the right eye shows an anomalous appearing disc with mild tilting and peripapillary atrophy (**e**). The optic disc photograph of the left eye showed subtle temporal optic disc pallor (**f**). Humphrey visual fields 30-2 of the right eye (**h**) and left eye (**g**) showed central depression. SD-OCT of the macula and nerve of the right eye (**i**) and left eye (**J**) were unremarkable. SD-OCT RNFL showed artifact inferiorly in the right eye consistent with the coloboma and showed normal thickness and contour in the left eye (**k**). The SD-OCT GCL analysis showed thickening in the inferonasal macula secondary to artifact in the right eye and mild thinning in the nasal macula in the left eye (**l**)

Four weeks after the intoxication, she presented to New England Eye Center. She had undergone intense physical therapy with near resolution of her neurological symptoms. However, she continued to have mild microphonia and felt that her vision remained persistently blurred bilaterally at distance and near. Best-corrected Snellen visual acuity was 20/30 in the right eye and 20/15 in the left eye. Pupils were reactive to light with no afferent pupillary defect in either eye. Color vision was intact by Hardy-Rand–Rittler pseudoisochromatic plates. Intraocular pressures were normal. Anterior segment examination of the right eye revealed a previously identified inferonasal colobomatous defect of the iris (Fig. 1a). The left eye was unremarkable (Fig. 1b). Ophthalmoscopy revealed a previously identified inferonasal chorioretinal coloboma in the right eye (Fig. 1c) and was unremarkable in the left eye (Fig. 1d). Examination of the optic nerve showed an anomalous appearing optic nerve with tilting and mild peripapillary atrophy in the right eye (Fig. 1e) and trace segmental pallor of the temporal optic nerve head in the left eye (Fig. 1f). Baseline Humphrey visual fields 30-2 showed mild central depression in the left (Fig. 1g) and the right (Fig. 1h) eyes. The SD-OCT (Cirrus; Carl Zeiss Meditec) of the macula in the right eye (Fig. 1i) and the left eye (Fig. 1j) eye were unremarkable. The SD-OCT retinal nerve fiber layer (RNFL) showed an abnormal contour with artifact inferiorly from the coloboma in the right eye and no thickening or thinning in the left eye (Fig. 1k). The SD-OCT ganglion cell layer (GCL) analysis showed thickening in the inferonasal macula secondary to artifact from the coloboma in the right eye and mild thinning in the nasal macula in the left eye (Fig. 1l). The patient was observed without treatment.

**Fig. 2:**
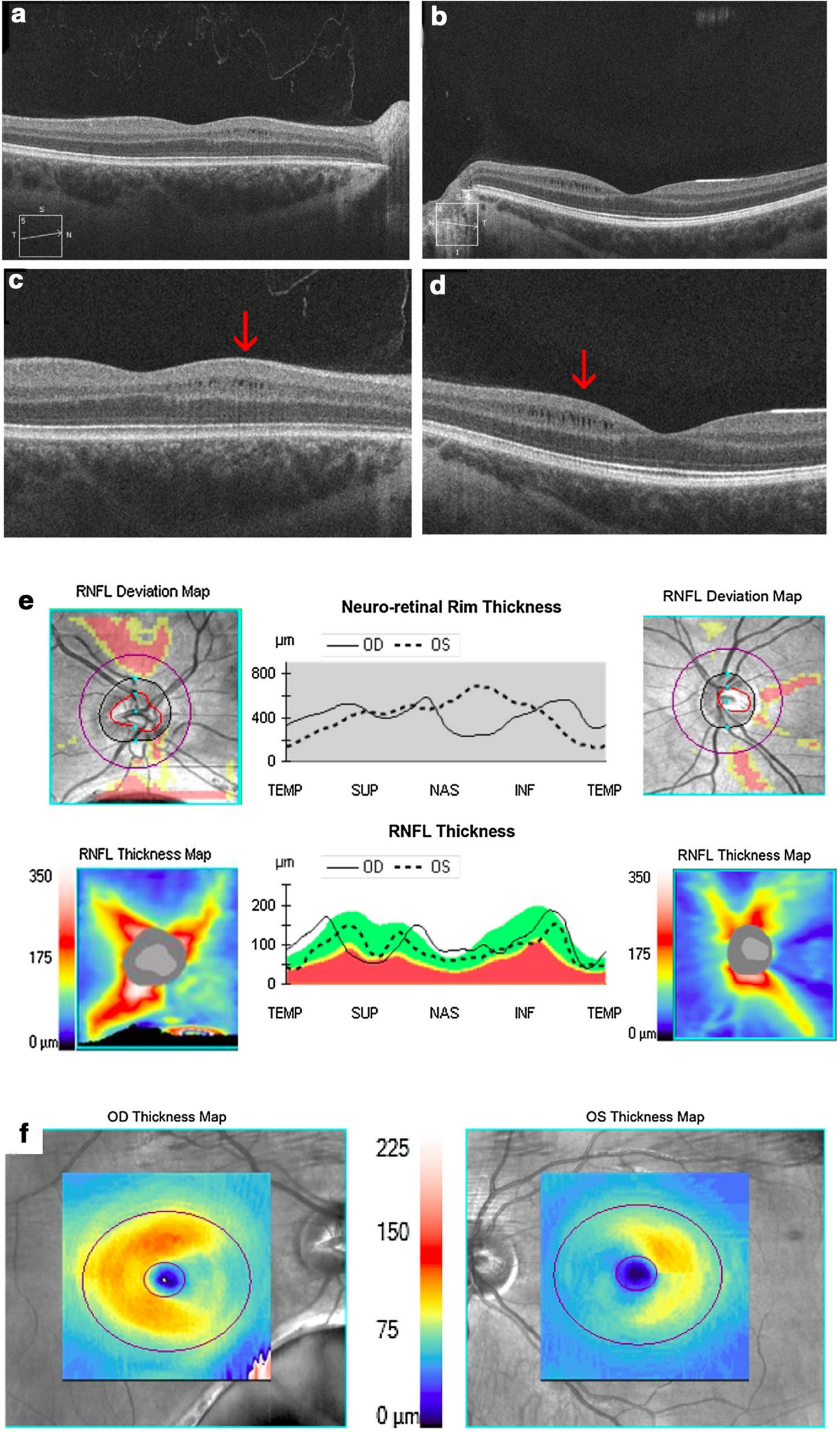
Findings 8 months post-intoxication. SD-OCT of the macula revealed ovoid shaped areas of hyporeflectivity in the nasal aspect of the macula in the right eye (**a**) and left eye (**b**). With greater magnification, these cysts appeared highly uniform and localized to the inner nuclear layer, consistent with INL microcysts in the right eye (**c**, *arrow*) and left eye (**d**, *arrow*). SD-OCT RNFL was unchanged from presentation in the right and left eyes (**e**). However, SD-OCT GCL analysis showed a wedge-shaped area of ganglion cell layer thinning in the nasal macula of both eyes, corresponding to the papillomacular bundle (**f**)

Eight months following the acute event of methanol intoxication, the patient reported that the vision had improved, although she had a persistent central “blind spot.” Best-corrected visual acuity remained 20/30 in the right and 20/15 in the left eye and her retinal examination was unchanged. SD-OCT of the macula showed a new finding of multiple retinal microcysts nasally in the right eye (Fig. 2a) and left eye (Fig. 2b). The cysts appeared highly uniform and localized to the inner nuclear (Fig. 2c, d). Although repeat SD-OCT of the RNFL was unchanged (Fig. 2e), the SD-OCT GCL now revealed bilateral wedge-shaped segmental defects of the papillomacular bundles (Fig. 2f).

## Discussion and conclusions

Although uncommon in the United States, methanol toxicity is a major public health concern in developing countries, and there have been increasing reports of tourist-related methanol toxicity while traveling to locations where home-brewed alcohol is consumed [1]. Acute methanol poisoning is potentially fatal and can have devastating visual consequences. Ophthalmologists should be familiar with its presentation. Fortunately, the patient was initially seen at a local hospital in Indonesia with providers familiar with the presenting features of methanol toxicity and received prompt treatment.

Prior OCT studies of methanol toxicity have shown peripapillary nerve fiber layer swelling acutely and diffuse retinal thinning chronically. However, inner nuclear layer microcysts have not previously been described [6]. Van Buren first described the histopathology of inner nuclear layer microcysts, from experimental optic nerve injury, and highlighted the extensive loss of the ganglion cells and cystic degeneration of the inner nuclear layer on the medial side of the fovea [7]. Since that time, microcystic inner nuclear layer abnormalities have been described in a variety of optic neuropathies including multiple sclerosis [8], neuromyelitis optica [5], hereditary optic neuropathies [9] and compressive optic pathway diseases [10]. Different theories have been put forth to explain these changes, including trans-synaptic retrograde degeneration, glial cell activation and mitochondrial dysfunction [7, 9, 10]. Microcysts should not be confused with cystoid macular edema or retinoschisis and are distinguishable on OCT. The former has a high degree of uniformity, characteristic shape and specific location within the INL whereas the latter tend to be larger, more irregular, and can localize to the outer plexiform layer as well. Further, INL microcysts are bilateral and the retina is not thickened [5]. Multimodal imaging of macular microcysts demonstrating unremarkable optical coherence tomography angiography of the superficial and deep retinal capillary plexuses and fluorescein angiography, suggest a nonvascular etiology such as Muller cell degeneration [5].

In this case, the pre-existing infero-nasal iris and optic nerve coloboma with peripapillary atrophy in the patient’s right eye is a potentially confounding factor and may lead to misinterpretation of OCT RNFL and GCL data. Nevertheless, despite severe systemic toxicity, the relative sparing of the optic nerve enabled characterization of the evolution of methanol optic nerve toxicity, suggesting early segmental retinal ganglion cell involvement corresponding to the selective loss of small caliber axons in the papillomacular bundle. This feature has been described in a variety of hereditary and acquired optic neuropathies characterized by oxidative stress and mitochondrial dysfunction, and reflects damage to the papillomacular bundle which provides central vision and is most vulnerable in energy-depleted states [4]. The finding of INL microcysts in this case suggests damage to retinal ganglion cells and may signify preferential loss off small caliber axons of the papillomacular bundle rich in mitochondria. These findings have not yet been described in methanol toxicity and may give insight into the evolution and pathophysiology of this devastating disease.

## Abbreviations

INL: inner nuclear layer
SD-OCT: spectral domain optical coherence tomography
RNFL: retinal nerve fiber layer
